# Th17-related genes PGAP1 and TMBIM1 serve as potential diagnostic and predictive biomarkers in systemic sclerosis: bioinformatic identification and murine model validation

**DOI:** 10.1007/s10067-026-07950-1

**Published:** 2026-01-31

**Authors:** Qian Li, Hanchao Li, Bomiao Ju, Zhiming Hao

**Affiliations:** Department of Rheumatology, the First Affiliated Hospital of Xi’an Jiaotong University, Xi’an, 710061 Shaanxi Province China

**Keywords:** Biomarkers, Machine learning, Single-cell sequencing, Systemic sclerosis, T helper 17 cells

## Abstract

**Background:**

T helper 17 (Th17) cells participate in all pathological stages of systemic sclerosis (SSc). Molecular markers associated with Th17 cells hold promise as therapeutic targets for SSc. This study aims to screen and validate key genes related to Th17 cells that have diagnostic and predictive value in SSc and to identify potential therapeutic targets for SSc.

**Methods:**

Candidate genes were identified by intersecting Th17 cell-dysregulated genes (TCDRGs) from single-cell RNA sequencing data (GSE292979) with bulk RNA sequencing data (GSE95065). Machine learning algorithms, receiver operating characteristic (ROC) curve analysis, and expression level validation were employed to further identify key Th17-related genes. Subsequently, the diagnostic and predictive capabilities of these Th17-related genes were validated using a nomogram model. Then, gene set enrichment analysis (GSEA), immune infiltration analysis, drug prediction, and molecular docking were conducted on the key genes. Finally, the expression of genes was confirmed in bleomycin (BLM)-induced SSc mice.

**Results:**

We identified 48 candidate genes from 472 TCDRGs and 1653 bulk DEGs. Using the Boruta feature selection algorithm and random forest (RF) model, two genes related to Th17 cells, PGAP1 and TMBIM1, were identified. PGAP1 and TMBIM1 exhibited exceptional discriminative efficiency with an AUC of 0.9852 in the nomogram model. Moreover, GSEA analysis revealed that both genes were co-enriched in pathways such as oxidative phosphorylation and the proteasome. Immune infiltration analysis indicated that PGAP1 and TMBIM1 were closely associated with five types of immune cells, exhibiting opposing correlation trends, such as with M1-type macrophages. Drug prediction analysis identified 45 potential drugs targeting PGAP1 and 36 targeting TMBIM1. Notably, Bafilomycin A1 was associated with PGAP1, while Ciglitazone was associated with TMBIM1, and both displayed stable binding conformations. Finally, in a BLM-induced mouse model, quantitative real-time PCR analysis and immunohistochemical experiments revealed upregulation of TMBIM1, whereas PGAP1 expression was downregulated in fibrotic skin and lung tissues. These results suggest the relevance of PGAP1 and TMBIM1 in fibrotic processes. However, further studies using human SSc samples are warranted to confirm their diagnostic and therapeutic potential.

**Conclusion:**

PGAP1 and TMBIM1 have been identified as key molecules associated with Th17 cells. They exhibit significant diagnostic and predictive value in SSc and show promise as therapeutic targets for SSc.

**Table:** 

**Key Points** • *SSc skin tissues exhibit significant changes in the composition of immune and non-immune cells.* • *PGAP1 and TMBIM1 have been identified as key molecules in SSc that are associated with Th17 cells, showing remarkable diagnostic accuracy.* • *PGAP1 and TMBIM1 exhibit a negative correlation in their expression patterns in SSc.*

**Supplementary Information:**

The online version contains supplementary material available at 10.1007/s10067-026-07950-1.

## Introduction

Systemic sclerosis (SSc) is an autoimmune disease characterized by immune system dysregulation, microvascular damage, and tissue fibrosis [1, 2]. Currently, the pathogenesis of SSc is not fully understood, and treatment options for SSc are limited. Traditional immunosuppressants exhibit limited efficacy and are associated with significant side effects [3, 4]. Moreover, due to the high non-specificity and complexity of early SSc clinical manifestations, diagnosis and treatment are often delayed until obvious skin involvement and/or clinically detectable visceral organ involvement occurs, at which time a definitive diagnosis is made. Nevertheless, by this stage, pathological changes in patients, such as microvascular remodeling, tissue fibrosis, or atrophy, are frequently irreversible, leading to the loss of the best treatment window. Consequently, developing and validating specific biomarkers detectable in the early stages of the disease and conducting in-depth analysis of its pathogenic mechanisms are key areas of focus for ongoing SSc research.

As the understanding of the immunopathological mechanisms of SSc has deepened, researchers have increasingly focused on specific immune cell subsets and their crucial roles in disease initiation and progression. Recent studies demonstrate that T helper 17 (Th17) cells and their related cytokines play a vital role in SSc pathogenesis [3, 5, 6]. The proportions of Th17 cells in the peripheral blood and lesion tissues of patients with SSc are significantly increased. Th17 cell levels significantly correlate with disease activity, suggesting their potential as biomarkers for evaluating disease severity and prognosis [7, 8]. Furthermore, Th17 cells increase cytokine levels, such as interleukin-17 (IL-17) and IL-23, activate fibroblasts, and promote extracellular matrix (ECM) deposition, ultimately leading to tissue fibrosis. IL-17 exacerbates the fibrosis process by inducing the secretion of other pro-fibrotic factors, including transforming growth factor-β (TGF-β) [9–11]. Th17 cells also play a crucial role in vascular lesions, causing endothelial cell injury and intensifying vascular inflammatory responses [12, 13]. Additionally, Th17 cells interact with other immune cells, such as macrophages and B cells, amplifying inflammatory responses and creating a vicious cycle that drives disease progression [8, 14]. Thus, Th17 cells may be a key driver that induces adaptive or pathological remodeling of the skin microenvironment, particularly affecting stromal cells like fibroblasts, keratinocytes, and endothelial cells. It should be noted that while some studies indicate that the proportion of Th17 cells in SSc tissues may vary, the abnormal functional status of these cells and their pivotal role in disease pathogenesis have been widely validated [4, 15]. Therefore, we aim to identify key genes closely associated with Th17 cell function by integrating single-cell and transcriptome data and then assess their diagnostic and predictive value in SSc.

This study utilizes public databases to systematically integrate single-cell and transcriptome data, aiming to screen for Th17 cell-related genes significantly associated with SSc and using R software for analysis. Subsequently, we conducted gene set enrichment analysis (GSEA) and immune infiltration analysis to explore the biological significance. Ultimately, the expression of Th17-related genes was detected in skin and lung samples from bleomycin (BLM)-induced mice. This research aims to provide a theoretical foundation and practical basis for elucidating the pathogenic mechanism of Th17 cells in SSc, optimizing diagnostic strategies, and identifying potential therapeutic targets.

## Materials and methods

### Data acquisition and processing

**Fig. 1:**
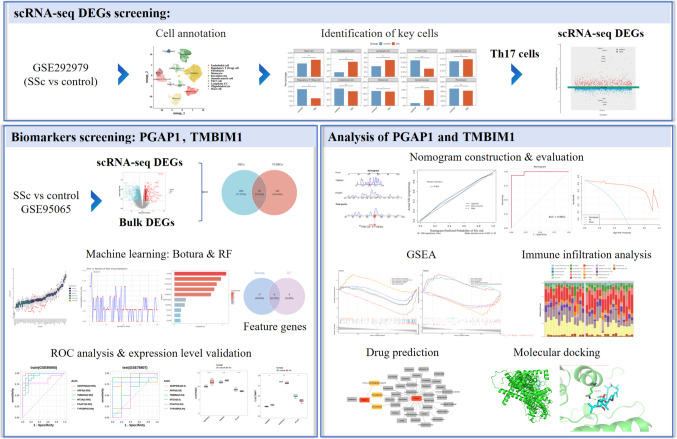
Flow chart of the study

The National Center for Biotechnology Information (NCBI) GEO database serves as an open-access platform for data (https://www.ncbi.nlm.nih.gov/geo/). The training dataset GSE95065 comprises 18 SSc skin tissue and 15 normal skin tissue samples. The validation dataset GSE76807 consists of 10 SSc skin tissue and 5 normal skin tissue samples. The single-cell dataset originates from the GSE292979 dataset and includes 12 SSc skin tissue samples and 3 normal skin tissue samples. The clinical information of the datasets is presented in the Supplementary Datas 1, 2, 3. The flow chart of this study is shown in Fig. 1.

### Identification of differentially expressed genes (DEGs)

To investigate the potential mechanisms underlying the role of Th17 cells in SSc, we isolated Th17 cell subsets from the single-cell dataset GSE292979 and conducted a DEG analysis to identify Th17 cell-dysregulated genes (TCDRGs). Thresholds for DEG screening were adjusted *p*-value <0.01 and |log2FC| ≥ 0.5.

In the transcriptome data analysis, we utilized the R package “limma” (version 3.54.0) to perform differential expression analysis between the SSc group and the control group using the training set GSE95065. The criteria for significance were set at |log2FC| > 0.5 and adjusted *p*-value < 0.01. The genes were defined as transcriptome differential genes (bulk DEGs).

TCDRGs were crossed with bulk DEGs to screen out candidate genes using the R package “VennDiagram” (version 1.7.3).

### Functional enrichment analysis and protein-protein interaction (PPI) of candidate genes

Functional enrichment analysis was conducted using the R package “clusterProfiler” (version 4.10.1) on the candidate genes, encompassing Gene Ontology (GO) and Kyoto Encyclopedia of Genes and Genomes (KEGG) pathway enrichment analysis. Selecting adjusted *p* <0.05, the biological processes (BPs), cellular components (CCs), molecular functions (MFs), and KEGG of candidate genes were analyzed. Additionally, protein-protein interaction (PPI) relationships among the candidate genes were analyzed using the STRING database (https://string-db.org). The interaction confidence threshold was set at > 0.15.

### Further screen candidate genes through machine learning analysis

Two machine learning algorithms, Boruta algorithm and Random Forest (RF) model, were performed for further selection from candidate genes. R package “Boruta” (version 8.0.0) was utilized to implement the Boruta algorithm, with candidate genes serving as input variables. All genes labeled as “Confirmed” were subsequently retained. Subsequently, we constructed the RF classification model for candidate genes using the R package “randomForest” (version 4.7.1.1, https://journal.r-project.org/articles/RN-2002-022/). Based on the optimal ntree, we constructed the RF model and ranked the genes according to their importance, as determined by the Gini index. Finally, we used the R package “VennDiagram” to identify the intersection between the “Confirmed” genes screened by the Boruta algorithm and the top 10 genes ranked by importance in the RF model.

### Receiver operating characteristic (ROC) curve analysis

For each candidate gene screened from machine learning analysis, we plotted the ROC curve and calculated the area under the curve (AUC) to assess the discriminatory power in differentiating SSc samples from control samples. Genes with AUC values greater than 0.7 in both datasets were selected for further analysis. To further investigate the consistency of expression patterns for candidate genes across the training set (GSE95065) and the validation set (GSE76807), we used the R package “rstatix” (version 0.7.2) and performed Wilcoxon rank-sum tests to compare expression differences between SSc and the control group (*p* < 0.05). Ultimately, genes demonstrating significant expression differences and consistent trends in both datasets were identified as key genes associated with Th17 cells.

### Construction and evaluation of the nomogram

We constructed a nomogram prediction model with the R package “rms” (version 6.5.0) using the training set GSE95065. We then drew a calibration curve to assess the agreement between the model’s predicted probabilities and actual observations. Additionally, we employed the Hosmer-Lemeshow (HL) goodness-of-fit test to calculate HL statistics and their corresponding *p*-values, thereby validating the calibration performance of the model. To further assess the clinical utility of this model across various thresholds, we utilized the R package “rmda” (version 1.6.1) to generate a decision curve analysis (DCA) plot. This allowed us to evaluate the net benefit of the model at different decision probabilities.

### Functional enrichment analysis of the Th17-related genes

Gene set enrichment analysis (GSEA): Utilizing the R package “psych” (version 2.2.9) and “clusterProfiler” (version 4.10.1), we conducted GSEA analysis, with the significance criterion for GSEA at *p* < 0.05 and a normalized enrichment score (NES) > 1. Finally, we selected the five pathways with the most significant correlations or visualizations.

Immune infiltration analysis: R package “CIBERSORT” (version 0.1.0) was used to evaluate the infiltration of immune cells in both the SSc and control groups, and the Wilcoxon rank-sum test using the R package “rstatix” (version 0.7.2) was conducted; *p* < 0.05 was considered differential immune cells. To further investigate the correlations between differential immune cells and Th17 cell-related genes, we calculated the Spearman correlation coefficients; the significance threshold was set at a correlation coefficient > 0.3 with *p* < 0.05.

Drug prediction: Drug Signatures Database (DSigDB; https://dsigdb.tanlab.org/DSigDBv1.0/) was used to explore potential therapeutic drugs for SSc. The prediction results were imported into the Cytoscape software (version 3.9.1) to construct the “drug-gene” interaction network and visualize it.

Molecular docking: Obtain the three-dimensional structure information of the protein from the Universal Protein Resource (UniProt, https://www.uniprot.org/) and obtain the small molecular 3D structure of candidate drugs from the PubChem database (https://pubchem.ncbi.nlm.nih.gov/). Subsequently, through CB-Dock2 online tools to analyze molecular docking (https://cadd.labshare.cn/cb-dock2/php/index.php), evaluate the combination of drugs and protein ability. The docking results with the lowest binding energy were selected, and the PyMOL software (version 3.1.1) was used for the visualization and structural analysis of the molecular docking results.

Chromosome localization and gene correlation analysis: Given that the occurrence of SSc may be closely related to genetic factors, in order to explore the distribution of selected genes on chromosomes, the R package “RCircos” (version 1.2.2) was used to draw their localization maps on chromosomes. In addition, to further analyze the interrelationships among selected genes in SSc, based on the expression data of all samples in the training set GSE95065, Spearman correlation analysis was conducted using the R package “psych” (version 2.2.9) to evaluate the expression correlations among selected genes.

### BLM-induced skin and pulmonary fibrosis

Fibrotic mouse lung and skin tissues induced by BLM were sourced from our previous studies. Six-week-old male ICR mice, free of specific pathogens, were procured from the Experimental Animal Center of the School of Medicine, Xi’an Jiaotong University. The mice were kept at 25 °C with a 12-h light/dark cycle, and they had free access to a standard chow diet and water. Before the experiments, the mice were acclimated in our facilities for 1 week.

BLM-induced skin fibrosis: Twelve mice were equally assigned into phosphate buffered saline (PBS) and BLM groups. The BLM group received a total of 0.1 mL of BLM (diluted with PBS to a concentration of 100 μg/0.1 mL, Sigma, St. Louis, USA) via subcutaneous injection once daily for 3 consecutive weeks. The normal control (NC) group received a subcutaneous injection of PBS.

BLM-induced pulmonary fibrosis: The mice were administered a single dose of BLM (2.5 mg/kg body weight in 0.05 mL of sterilized PBS) via intratracheal instillation to induce pulmonary fibrosis on day 0. Three mice were sacrificed, respectively, at 1, 3, 5, 7, 14, and 28 days post-BLM treatment. The NC group received a PBS intratracheal instillation.

### Reverse transcription quantitative real-time PCR (qRT-PCR)

Total RNA was extracted from skin and lung tissues with Trizol reagent (Thermo, Life Technologies, Carlsbad, CA) and quantified via NanoDrop 2000 spectrophotometer (Thermo Fisher Scientific, Carlsbad, CA). Reverse transcription was performed with a PrimeScript™ RT reagent Kit (TaKaRa, Dalian, China). The relative abundance of each mRNA in the sample was determined using qRT-PCR with the corresponding primers (Table 1) and the SYBR® Premix Ex Taq™II (TaKaRa, Dalian, China) on an iQ™ Multicolor Real-time PCR Detection System (Bio-Rad, Hercules, CA). Data were analyzed using the ΔΔCT method, and β-actin served as an internal control.

**Table 1:** qRT-PCR primers

Gene	Species	Forward	Reverse
β-actin	mouse	5′-CCTCTATGCCAACACAGTGC-3′	5′-CACACAGAGTACTTGCGCTC-3′
TMBIM1	mouse	5′-CTGGCTCCACATGGTCTATGC-3′	5′-GTGTAGATCTGTAGGGCGCC-3′
PGAP1	mouse	5′-CTTAGTCCTGACCCATGCAAAC-3′	5′-GGAATGATCACAAAACACGG-3′

### Immunohistochemical (IHC) experiments

IHC was performed on BLM-induced fibrotic skin tissues (3 weeks after BLM subcutaneous injection) and lung tissues (14 days after BLM intratracheal instillation) using the two-step IHC detection reagent (Beijing ZhongshanJinqiao Biotechnology Co., Ltd, China). Paraffin-embedded tissue sections, each measuring 5 μm thick, were dewaxed and hydrated. Subsequently, antigen retrieval was performed using a Tris-EDTA buffer (pH 9.0) under microwave heating. Next, 3% H_2_O_2_ was employed to block endogenous peroxidase, and the sections were incubated overnight at 4 °C with a rabbit anti-human TMBIM1 antibody (1:200, Proteintech, China) and a rabbit anti-human PGAP1 antibody (1:200, Proteintech, China). On the following day, 100 μL of reaction enhancer solution was added, and the sections were incubated at 37 °C for 20 min. After washing with PBS buffer, the sections were incubated with goat anti-rabbit IgG polymer for another 20 min. DAB color development, hematoxylin counterstaining, and mounting with neutral gum were performed. Immunohistochemical images of the tissue sections were acquired using a light microscope (Olympus BX51, Olympus, Tokyo, Japan) equipped with a DP70 digital camera.

### Statistical analysis

For the mRNA expression analysis, GraphPad Prism software version 6 (GraphPad Software Inc., La Jolla, CA, USA) was used and data are expressed as the mean ± standard deviation (SD). Differences between groups were assessed using an unpaired Student’s *t* test. All the other analyses were conducted using R software (version 4.2.2). The statistical assessment of differences between groups was conducted using the Wilcoxon rank sum test. *p* < 0.05 was considered to be statistically significant.

## Results

### The proportion of ten cell types in the SSc skin tissue undergoes alterations

**Fig. 2:**
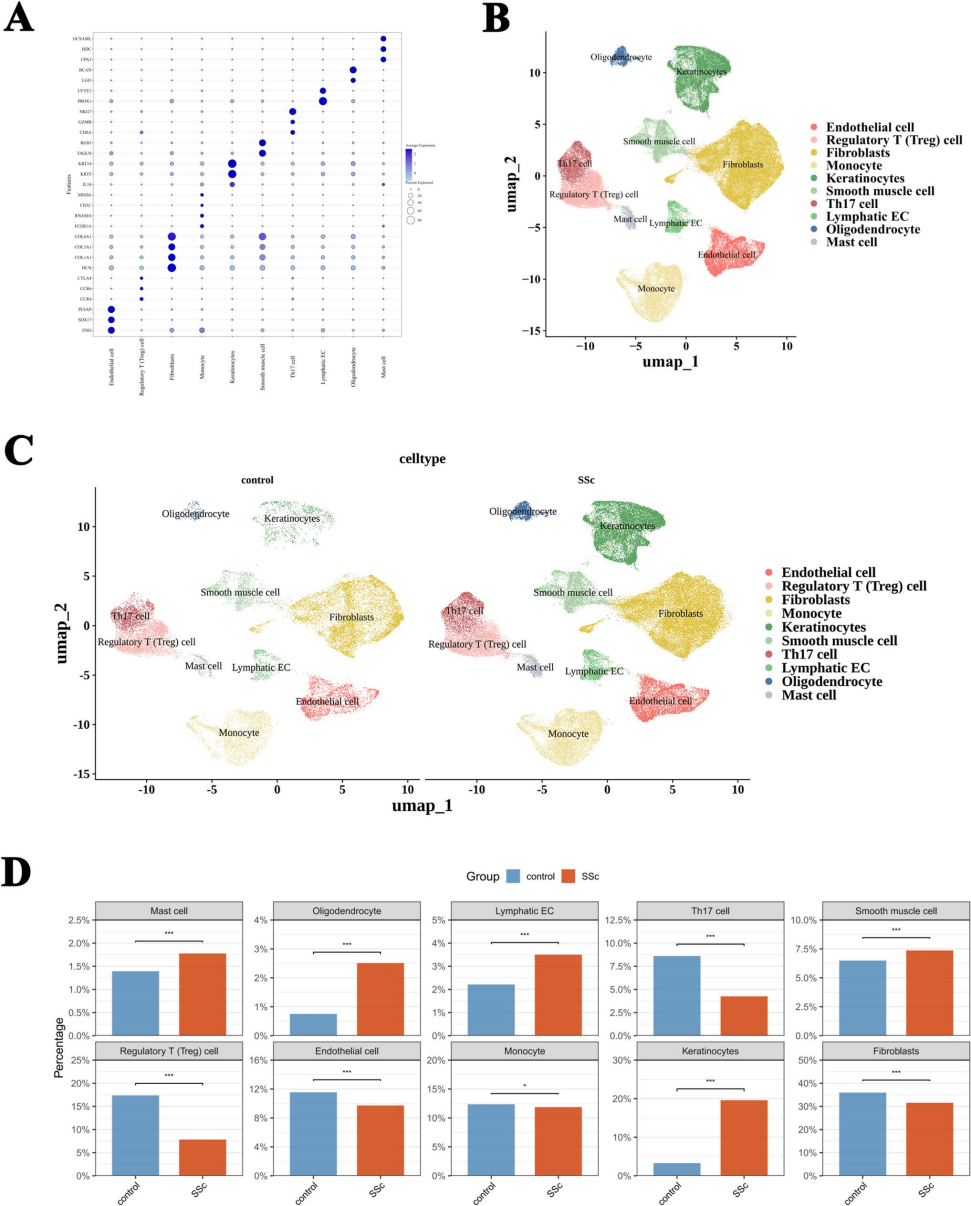
Dimensional reduction analysis of skin tissue scRNA-seq data from SSc. **A** The expression of marker genes in different cell types using bubble plots. **B** Distribution of different cell types in UMAP. **C** UMAP of skin tissue scRNA-seq divided by SSc and control cohorts. **D** Ten annotated cells showed significant differences between the SSc and control groups. *p < 0.05, **p < 0.01 vs control group

The single-cell dataset GSE292979 was utilized to evaluate changes in infiltrating cells within SSc skin tissues. According to the expression patterns of characteristic genes (Fig. 2A), we annotated 16 cell clusters and identified 10 major cell types (Fig. 2B): endothelial cells, regulatory T cells (Tregs), monocytes, fibroblasts, keratinocytes, smooth muscle cells, Th17 cells, lymphatic endothelial cells (lymphatic ECs), mast cells, and oligodendrocytes. Further comparison of the proportions of these cell types between SSc and control groups revealed significant differences in the 10 annotated cell types (Fig. 2C, D). Specifically, the infiltration ratios of mast cells, oligodendrocytes, lymphatic endothelial cells, smooth muscle cells, and keratinocytes were significantly increased in the SSc group, whereas those of endothelial cells, Tregs, monocytes, fibroblasts, and Th17 cells were significantly decreased. Our study demonstrates that the cellular makeup of the immune microenvironment in the skin of SSc patients undergoes significant remodeling. Although the proportion of Th17 cells has relatively decreased, their pivotal role in driving major pathological processes, such as fibrosis, remains undiminished. This phenomenon may be attributed to the dynamic modulation of the microenvironment or changes in cellular states. With this objective, we further analyzed the differentially expressed genes in Th17 cells to reveal changes in their functional status and identify potential diagnostic biomarkers.

### Identification of DEGs and candidate genes associated with Th17 cells

**Fig. 3:**
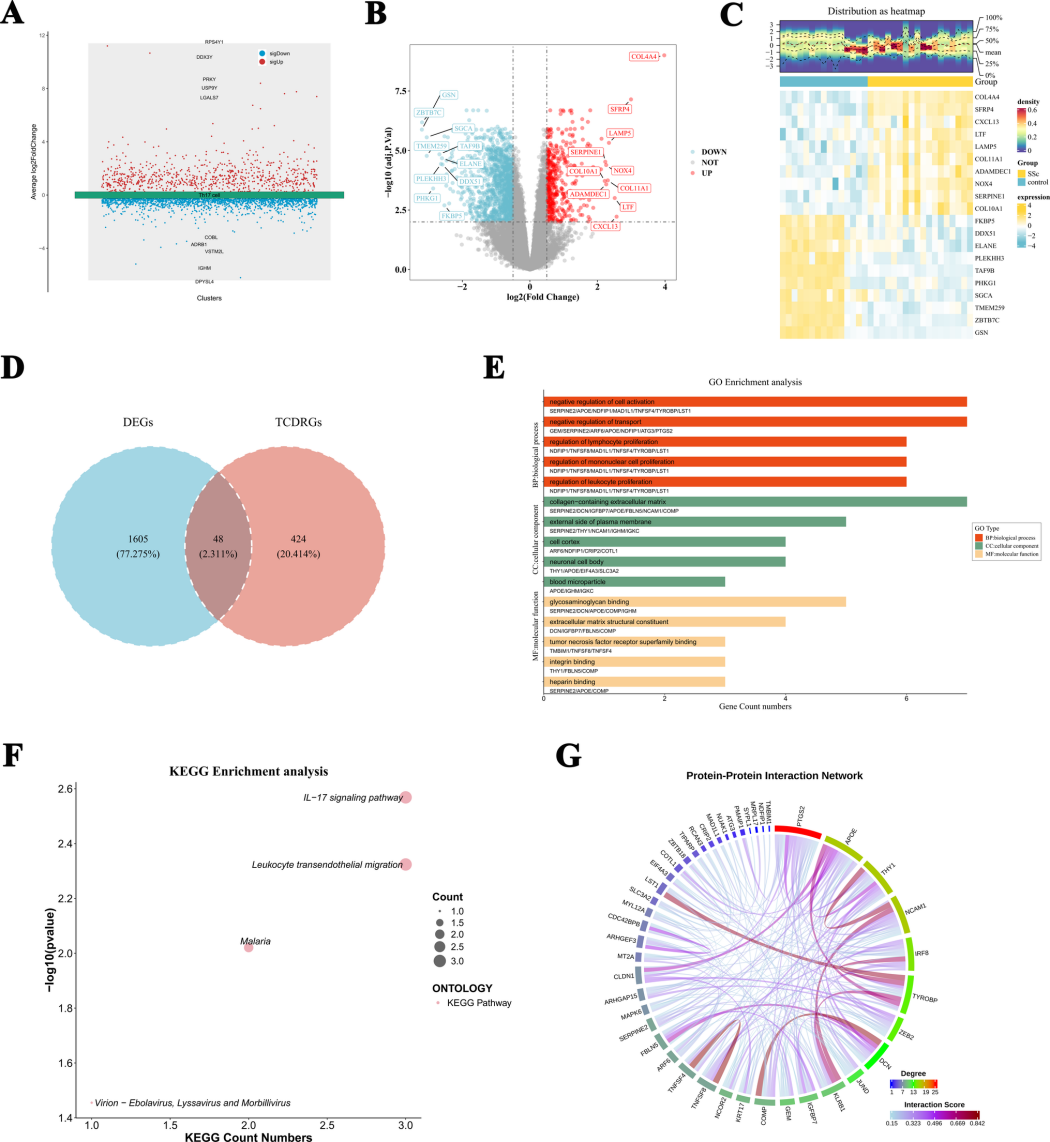
Identification of DEGs and candidate genes associated with Th17 cells. **A** Th17 cell-dysregulated genes (TCDRGs) between the SSc and control groups in the GSE292979 scRNA-seq dataset. **B**, **C** Bulk DEGs in the GSE95065 RNA sequencing dataset. **D** Forty-eight candidate genes were defined by taking the intersection of the TCDRGs and bulk DEGs. **E** GO enrichment analysis of candidate genes. **F** KEGG pathway enrichment analysis of candidate genes. **G** The PPI network analysis of candidate genes

We screened out 472 Th17 cell-dysregulated genes (TCDRGs) between the SSc and control groups in the GSE292979 scRNA-seq dataset (Fig. 3A). Among these TCDRGs, the top five genes significantly upregulated in SSc were RPS4Y1, DDX3Y, PRKY, USP9Y, and LGALS7, whereas significantly downregulated were DPYSL4, IGHM, VSTM2L, ADRB1, and COBL. In the GSE95065 bulk RNA sequencing dataset, we identified a total of 1653 bulk DEGs between the SSc and control groups, with 1223 genes upregulated and 430 genes downregulated in the SSc samples (Figure 3B, C). We identified 48 overlapping genes by taking the intersection of the 472 scRNA-seq DEGs and 1653 bulk DEGs, which were defined as candidate genes (Fig. 3D).

To investigate the potential roles of 48 candidate genes, we conducted GO enrichment analysis and identified 497 significant enrichment pathways, comprising 437 biological processes (BPs), 30 cellular components (CCs), and 30 molecular functions (MFs) (Fig. 3E and Supplementary Data 4). In the BP dimension, the candidate genes are predominantly enriched in processes such as negative regulation of cell activation, negative regulation of transport, and regulation of lymphocyte, mononuclear cell, and leukocyte proliferation. In the CC dimension, the enrichment pathways encompass collagen-containing extracellular matrix, the external side of the plasma membrane, cell cortex, neuronal cell body, and blood microparticles, among others. In the MF dimension, significant pathways include glycosaminoglycan binding, extracellular matrix structural components, tumor necrosis factor receptor superfamily binding, integrin binding, and heparin binding, among others. Further KEGG pathway enrichment analysis was conducted, revealing four significant pathways: the IL-17 signaling pathway, leukocyte transendothelial migration, malaria, and viral particle-related pathways (including Virion-Ebolavirus, Lyssavirus, and Morbillivirus) (Fig. 3F and Supplementary Data 5). The PPI network demonstrated complex interaction relationships among the proteins encoded by the candidate genes. Notably, DCN and TYROBP exhibit a high degree of connectivity within the network and may play pivotal regulatory roles in the pathogenesis and progression of SSc (Fig. 3G). Our results indicate that candidate genes in SSc may collectively regulate the onset and progression of the disease through involvement in various biological processes, including immune modulation, extracellular matrix reorganization, and inflammatory signaling cascades.

### PGAP1 and TMBIM1 have been identified as key Th17-related genes

**Fig. 4:**
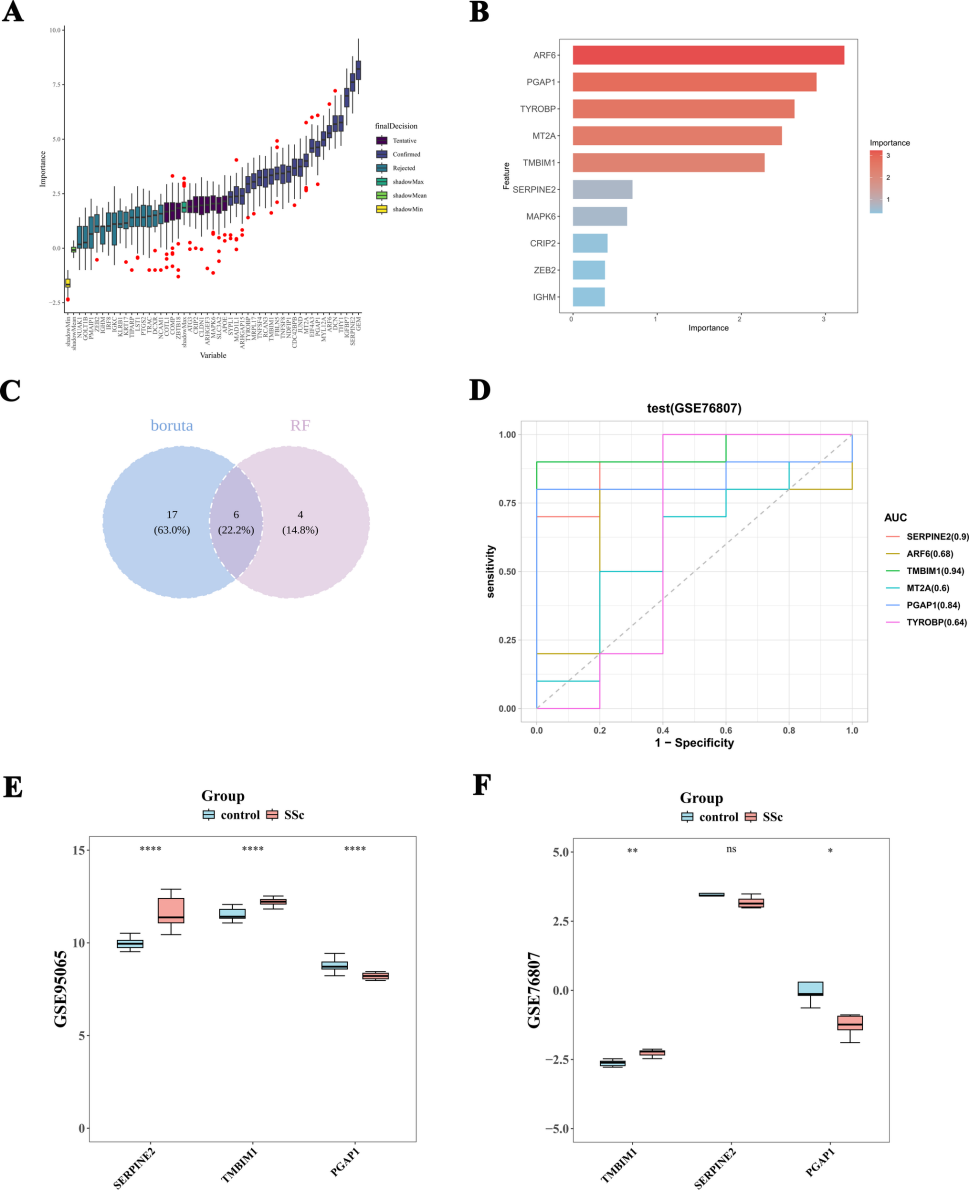
PGAP1 and TMBIM1 have been identified as key Th17-related genes. **A** Boruta algorithm and **B** RF model of candidate genes. **C** Six characteristic genes were defined by taking the intersection of the Boruta algorithm and the RF model. **D** ROC analysis of 6 characteristic genes. **E**, **F** SERPINE2, PGAP1, and TMBIM1 expression in both the GSE95065 training set and the GSE76807 validation set

The Boruta algorithm and RF model were utilized to identify key Th17-related genes. The Boruta algorithm identified 23 genes with confirmed importance, with GEM and SERPINE2 exhibiting significantly higher scores compared to the others (Fig. 4A). The RF model selected the top 10 genes ARF6, PGAP1, TYROBP, MT2A, TMBIM1, SERPINE2, MAPK6, CRIP2, ZEB2, and IGHM (Fig. 4B). By intersecting the confirmed genes from the Boruta algorithm with the top 10 genes from the RF model, six overlapping genes were identified: SERPINE2, ARF6, TMBIM1, MT2A, PGAP1, and TYROBP (Fig. 4C).

To assess the diagnostic potential of the six characteristic genes further, we conducted ROC analysis in both the GSE95065 training set and the GSE76807 validation set. The results revealed high AUC values for SERPINE2 (0.996, 0.900), TMBIM1 (0.952, 0.940), and PGAP1 (0.933, 0.840) in both datasets (Fig. 4D, E). Comparison of SERPINE2, PGAP1, and TMBIM1 expression between the SSc and control groups showed significant differences in TMBIM1 and PGAP1 expression levels across both datasets, with consistent trends (Fig. 4F, G). Specifically, TMBIM1 expression was significantly upregulated, while PGAP1 expression was downregulated in SSc samples.

Consequently, PGAP1 and TMBIM1 were identified as key Th17-related genes in SSc.

### The nomogram model based on PGAP1 and TMBIM1 exhibits remarkable diagnostic accuracy

**Fig. 5:**
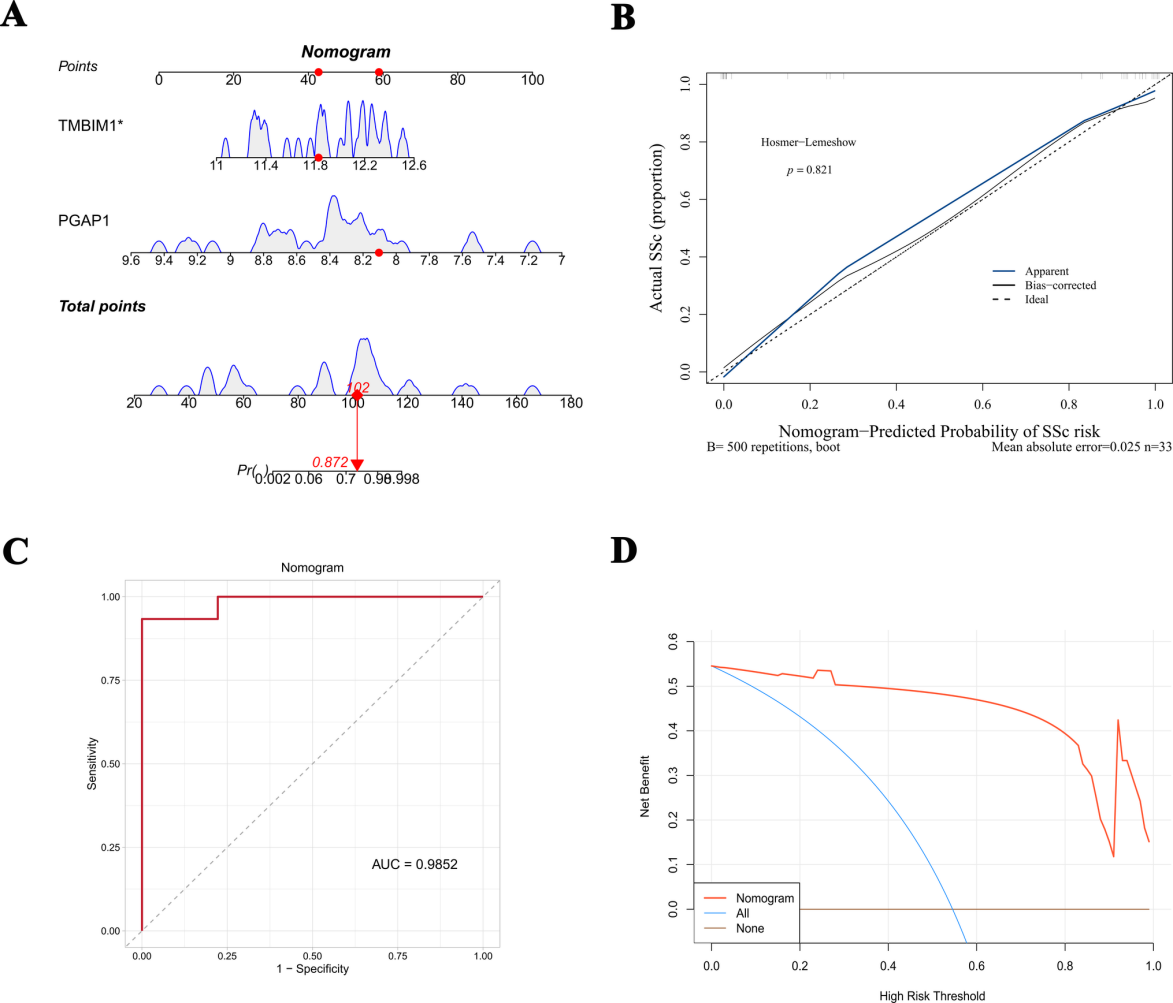
The nomogram model based on PGAP1 and TMBIM1 exhibits remarkable diagnostic accuracy. **A** PGAP1 and TMBIM1 correspond to a predicted probability of illness of 87.2% in a nomogram model. **B** High consistency between the predicted and actual occurrence probabilities using a calibration curve. **C**, **D** The ROC curve validated the nomogram’s high accuracy in distinguishing SSc from control samples

A nomogram for predicting SSc was developed using PGAP1 and TMBIM1 as predictors. The model assigns a score to each gene based on its expression level, with the total score representing the sum of the scores for PGAP1 and TMBIM1. A total score of 102 for PGAP1 and TMBIM1 corresponds to a predicted probability of illness of 87.2% (Fig. 5A). The accuracy of the prediction model was evaluated using a calibration curve (Fig. 5B). The results indicated a high consistency between the predicted and actual occurrence probabilities. The HL goodness-of-fit test result was *p* = 0.821, indicating no statistical difference and confirming the model’s good calibration ability. The ROC curve further validated the model’s discriminative efficacy, with an AUC value of 0.9852, demonstrating the nomogram’s high accuracy in distinguishing SSc from control samples (Fig. 5C). Additionally, the DCA results showed that within most risk threshold ranges, the net benefit of the model was higher than that of the “All intervention” and “No intervention” scenarios, despite slight fluctuations in the net benefit curve when the threshold exceeded 0.8. Overall, the model demonstrates good clinical application value (Fig. 5D). In conclusion, the nomogram based on PGAP1 and TMBIM1 demonstrates excellent accuracy and practicality for predicting SSc and supporting clinical decisions.

### PGAP1 and TMBIM1 may play pivotal roles in the pathogenesis of SSc via various metabolic and disease-associated pathways

**Fig. 6:**
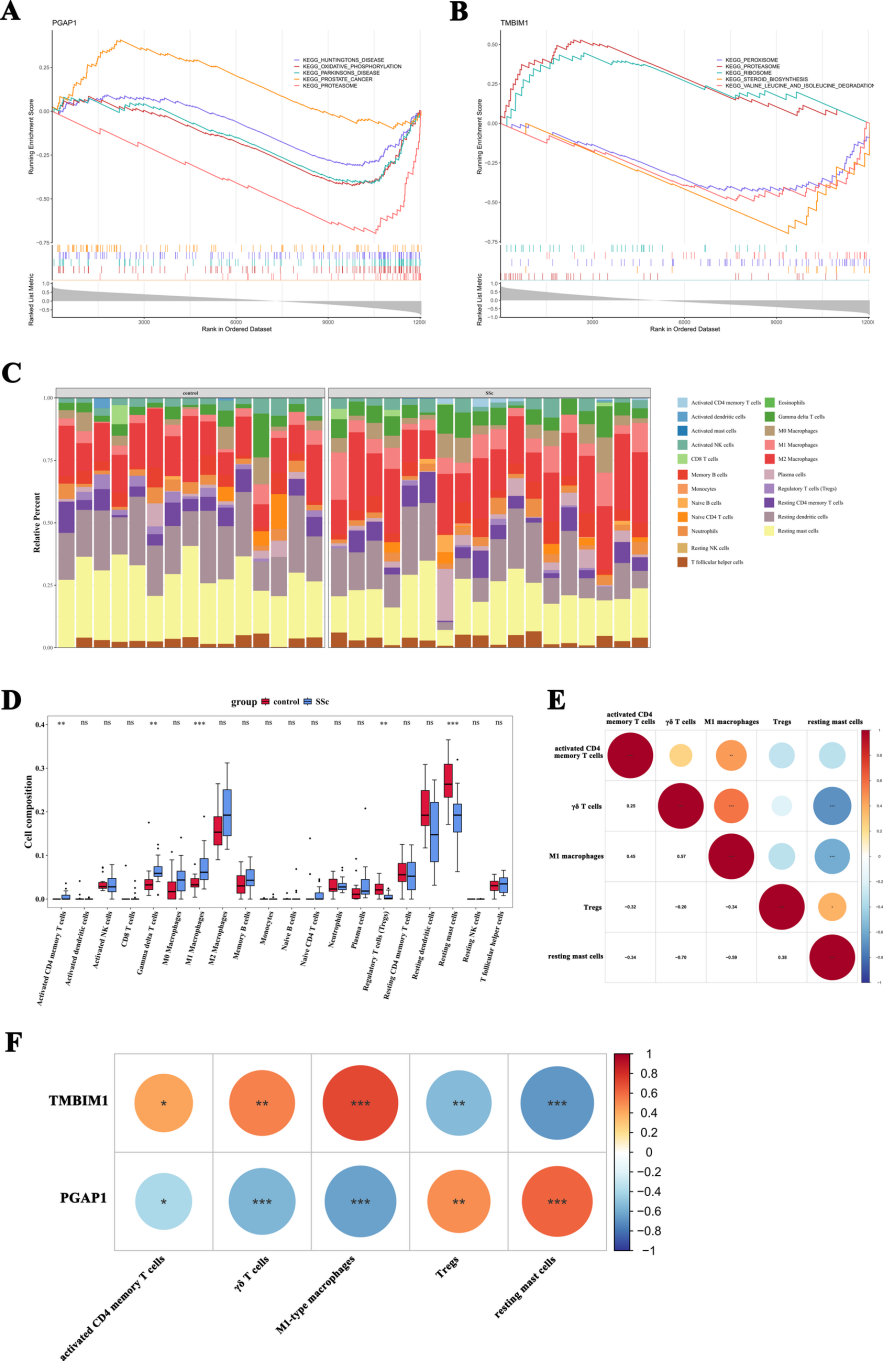
GSEA and immune infiltration analysis of Th17-related genes. **A**, **B** GSEA analysis of PGAP1 and TMBIM1. **C** The composition of 22 immune cell types in both SSc and control groups. **D** Five immune cell types showed significant differences between the SSc and control groups. **E** Correlation analysis of the five immune cell types. **F** The correlation between PGAP1 and TMBIM1 as well as the five immune cell types. *p < 0.05, **p < 0.01

GSEA analysis indicated that PGAP1 and TMBIM1 were significantly enriched in 45 and 36 pathways, respectively. Specifically, 20 pathways were jointly enriched by both PGAP1 and TMBIM1, including key pathways like proteasome and oxidative phosphorylation (Figs. 6A, B and Supplementary Datas 6-7). PGAP1 was particularly enriched in pathways related to Parkinson’s disease, Huntington’s disease, and prostate cancer. In contrast, TMBIM1 was involved in steroid biosynthesis, peroxisome function, valine, leucine, and isoleucine degradation, as well as ribosome pathways. The GSEA results suggest that PGAP1 and TMBIM1 may play crucial roles in the onset and progression of SSc via multiple metabolic and disease-related signaling pathways, potentially exhibiting functional heterogeneity and complementary effects.

### PGAP1 and TMBIM1 are associated with differential immune cell populations in SSc

Utilizing the GSE9506 training set, we analyzed the composition of 22 immune cell types in both SSc and control groups. The results revealed high proportions of M2 macrophages, resting dendritic cells and resting mast cells in both groups (Fig. 6C). Further analysis identified significant differences in five immune cell types between the SSc and control groups (Fig. 6D). In the SSc group, activated CD4 memory T cells, γδ T cells, and M1-type macrophages were upregulated, whereas Tregs and resting mast cells were downregulated. Correlation analysis of these five immune cell types showed that γδ T cells were positively correlated with M1-type macrophages and negatively correlated with resting mast cells (Fig. 6E). M1-type macrophages were positively correlated with activated CD4 memory T cells and negatively correlated with resting mast cells. Resting mast cells were positively correlated with Tregs.

Subsequently, we analyzed the correlation between PGAP1 and TMBIM1 as well as differential immune cells (Fig. 6F). However, the correlation patterns between PGAP1 and TMBIM1 were opposite. TMBIM1 was positively correlated with activated CD4 memory T cells, γδ T cells, and M1-type macrophages and exhibited a significant negative correlation with Tregs and resting mast cells. PGAP1, in contrast, was negatively correlated with activated CD4 memory T cells, γδ T cells, and M1-type macrophages while exhibiting a significant positive correlation with Tregs and resting mast cells. These findings align with the observed upregulation of TMBIM1 and downregulation of PGAP1 in SSc samples, suggesting that PGAP1 and TMBIM1 may play crucial roles in the immune microenvironment remodeling of SSc.

### Identifies potential candidate drugs for PGAP1 and TMBIM1

**Fig. 7:**
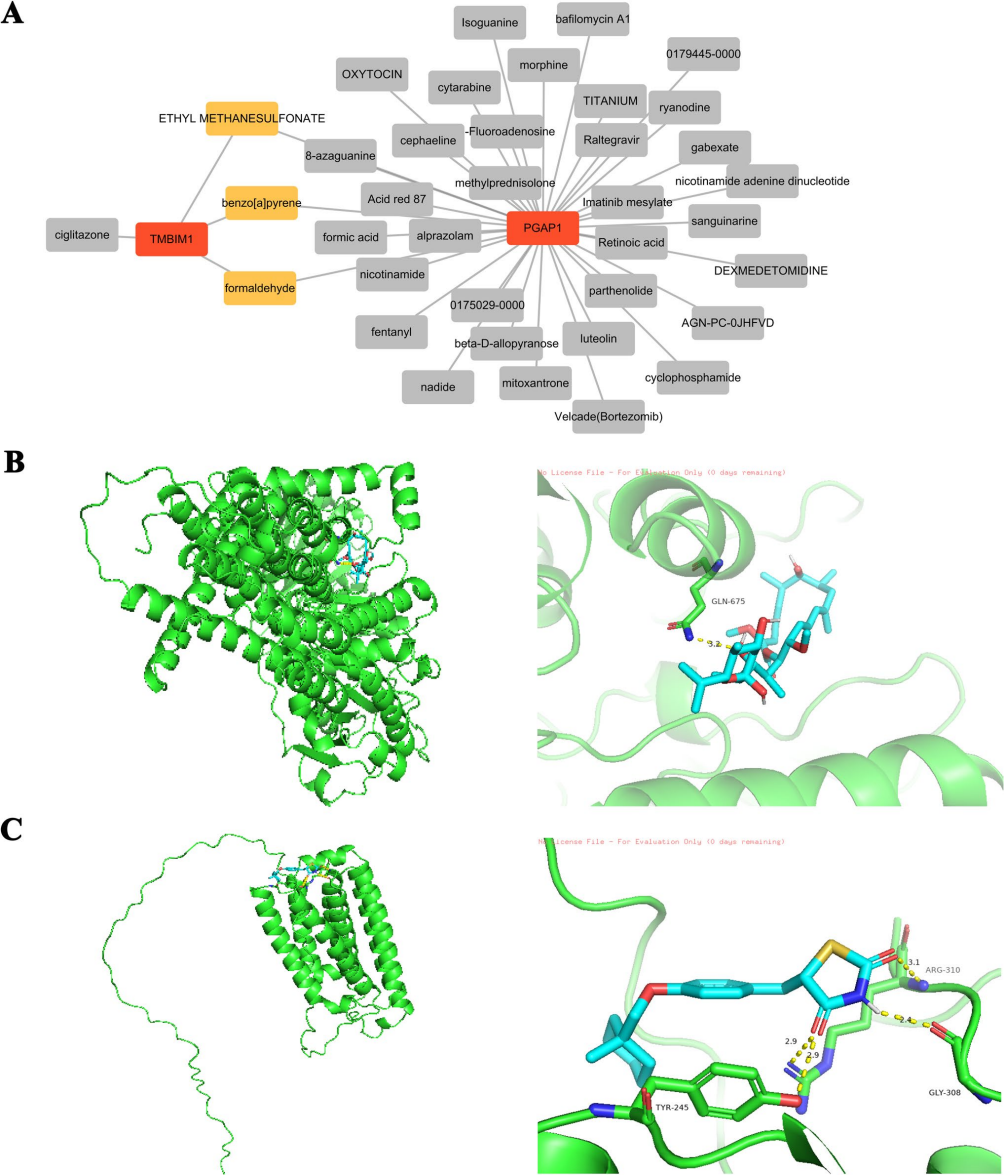
Drug prediction and molecular docking. **A** Drug prediction results indicate that PGAP1 and TMBIM1 are predicted to have 36 and 4 potential targeted drugs. **B**, **C** Molecular docking results revealed a stable binding conformation between PGAP1 and Bafilomycin A1, TMBIM1, and Ciglitazone

The drug prediction results indicate that PGAP1 and TMBIM1 have 36 and 4 potential targeted drugs, respectively (Fig. 7A). Specifically, ethyl methanesulfonate, formaldehyde, and benzo[a]pyrene were predicted to target both biomarkers simultaneously. However, the binding energies of these three drugs exceed −5 kcal/mol, failing to meet the binding stability criteria for molecular docking. Among the other predicted drugs, Bafilomycin A1, a V-ATPase inhibitor, may be involved in endoplasmic reticulum sorting and vesicle transport of GPI-AP regulated by PGAP1. Ciglitazone, an agonist of PPARγ, is closely associated with the adipogenesis pathway regulated by TMBIM1. Based on the aforementioned literature, Bafilomycin A1-PGAP1 and Ciglitazone-TMBIM1 pairs were selected for molecular docking analysis. During the docking process, the PDB files representing conformations with binding energies of −12.6 kcal/mol (PGAP1-Bafilomycin A1) and −6.7 kcal/mol (TMBIM1-Ciglitazone) were selected. Molecular docking results revealed a stable binding conformation between PGAP1 and Bafilomycin A1, with GLN-675 forming a hydrogen bond with the drug at a distance of 3.2 Å (Fig. 7B). The docking results for TMBIM1 and Ciglitazone also indicated a stable binding state, involving amino acid residues TYR-245 (forming two hydrogen bonds at 2.9 Å), CLE-308 (2.4 Å), and ARG-310 (3.1 Å) (Fig. 7C). The drug exhibited good binding activity with TMBIM1. These results suggest that Bafilomycin A1 and Ciglitazone could potentially serve as regulatory drugs for PGAP1 and TMBIM1, offering a new candidate for targeted therapy in SSc.

### PGAP1 and TMBIM1 exhibit a negative correlation in their expression patterns in SSc

**Fig. 8:**
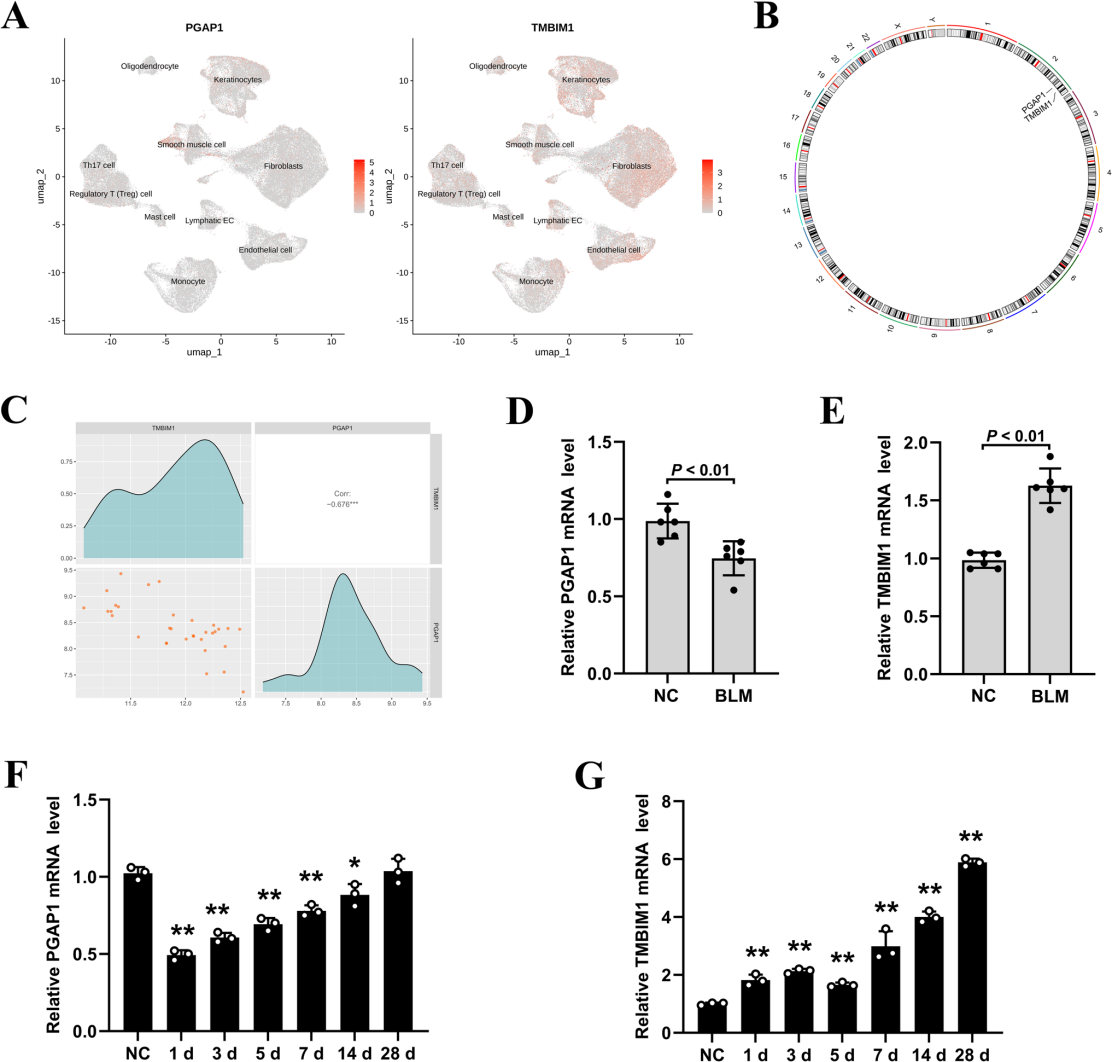
PGAP1 and TMBIM1 exhibit a negative correlation in their expression patterns in SSc. **A** PGAP1 and TMBIM1 expression in skin cells originates from the GSE292979 dataset. **B** Chromosome localization analysis revealed PGAP1 and TMBIM1 reside on human chromosome 2. **C** PGAP1 and TMBIM1 showed a significant negative correlation using correlation analysis. **D** The expression of PGAP1 mRNA decreased in the fibrotic skin tissues of mice induced by BLM. **E** The expression of TMBIM1 mRNA increased in the fibrotic skin tissues of mice. **F** PGAP1 mRNA expression in mice fibrotic lung tissues. **G** TMBIM1 mRNA expression in mice fibrotic lung tissues. *p < 0.05, **p < 0.01 vs NC group

The expression pattern of TMBIM1 and PGAP1 in the single-cell dataset GSE292979 was examined. As shown in Fig. 8A, PGAP1 was predominantly expressed in keratinocytes and smooth muscle cells, with low expression in fibroblasts and T cells. TMBIM1 was expressed in all cell subsets. Subsequent chromosomal localization analysis and correlation analysis in the training set GSE95065 demonstrated that both PGAP1 and TMBIM1 reside on human chromosome 2 (Fig. 8B) with a significant negative correlation between PGAP1 and TMBIM1 (Fig. 8C). We then examined PGAP1 and TMBIM1 mRNA expression in BLM-induced fibrotic skin tissue (3 weeks after BLM subcutaneous injection) and lung tissues at various time points (1, 3, 5, 7, 14, and 28 days after BLM intratracheal instillation). In mice fibrotic skin tissues, qRT-PCR demonstrated that PGAP1 mRNA expression decreased (Fig. 8D) while TMBIM1 expression increased compared with normal control (NC) (Fig. 8E). In the lung tissues of mice, PGAP1 levels significantly decreased following BLM treatment and gradually returned to normal by day 28 (Fig. 8F). Meanwhile, TMBIM1 expression gradually increased after BLM treatment (Fig. 8G), aligning with the observed downregulation of PGAP1 and upregulation of TMBIM1 in the set GSE95065. These findings suggest that PGAP1 and TMBIM1 may play a mutual regulatory or functionally antagonistic role in the pathogenesis of SSc.

### PGAP1 and TMBIM1 proteins are dysregulated in the skin and lung tissues of BLM-induced SSc mice

**Fig. 9:**
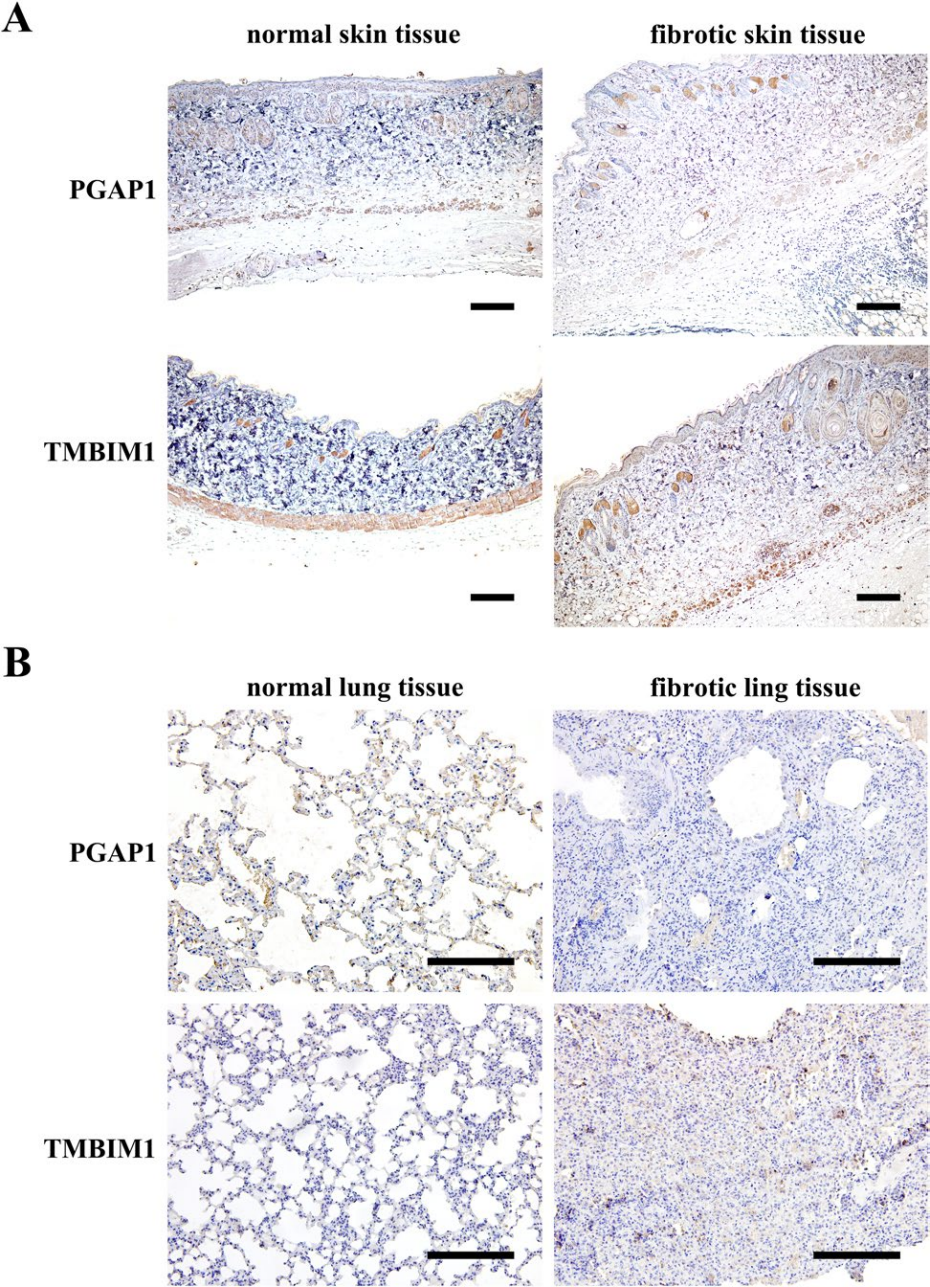
The PGAP1 and TMBIM1 expression in mice fibrotic skin and lung tissue. **A** PGAP1 expression was decreased while TMBIM1 increased in fibrotic skin of BLM-induced fibrotic skin. **B** In BLM-induced fibrotic lung tissues, PGAP1 expression was decreased and TMBIM1 expression was increased. Scale bars = 200 μm

To validate the bioinformatics analysis and mRNA results, we conducted IHC on the skin and lung tissues of BLM-induced SSc mice. As shown in Fig. 9, PGAP1 was mainly expressed in keratinocytes and skin appendages, and its expression decreased in the fibrotic skin and lung tissues of BLM-induced SSc mice. In contrast, we observed an increased expression of TMBIM1 in the fibrotic skin and lung tissues, especially in keratinocytes and extracellular matrix regions. These findings are consistent with the trends of increased TMBIM1 expression and decreased PGAP1 expression observed in the transcriptome data analysis, further suggesting the potential involvement of both proteins in the pathogenesis of SSc.

## Discussion

Th17 cells are involved in three pathological processes of SSc: immune system dysregulation, microvascular damage, and extensive fibrosis. In this study, we utilized public databases to investigate the potential molecular mechanisms and diagnostic value of Th17-related genes in SSc. Initially, single-cell analysis demonstrated a significant reduction in the number of Th17 cells in SSc skin tissues. By using bioinformatics techniques, we identified two Th17-associated genes, PGAP1 and TMBIM1, which exhibited high predictive accuracy and substantial potential for clinical application in SSc. Further functional analysis revealed that PGAP1 and TMBIM1 exhibit functional complementarity in SSc and may regulate several key metabolic and inflammatory signaling pathways. Finally, we confirmed their expression in fibrotic tissues. PGAP1 expression decreased in mouse fibrotic skin and lung tissues, while TMBIM1 mRNA levels increased in both fibrotic skin and lung tissues.

In our study, PGAP1 and TMBIM1 are linked to changes in the immune microenvironment of SSc and may play a role in immune dysregulation and fibrosis by modulating Th17 cell function. TMBIM1 is a transmembrane protein [16]; as a pH-sensitive Ca2^+^ channel, TMBIM1 can trigger membrane permeability and cell death in response to lysosomal stress, implying context-specific regulation of the cell survival/death balance [17, 18]. Additionally, TMBIM1 facilitates lysosomal degradation of Toll-like receptor 4 (TLR4), modulates innate immune signaling, and potentially reshapes the immune microenvironment [19, 20]. Although no studies have investigated the role of TMBIM1 in SSc, previous studies have demonstrated that disrupted lysosomal Ca^2^⁺ homeostasis is closely associated with immune cell activation [21]. Abnormal activation of Th17 cells, myeloid-derived suppressor cells (MDSCs), and macrophages has been confirmed as a key factor in the immunopathogenesis of SSc [13]. Additionally, abnormal activation of the TLR4 signaling pathway is implicated in the fibrotic process of SSc [22]. In our study, we found significant upregulation of TMBIM1 in SSc, with enrichment pathways predominantly associated with fat metabolism, cell survival, and immune regulation. Therefore, we propose that upregulation of TMBIM1 promotes immune cell infiltration and activation (e.g., Th17, MDSC, and macrophages) by modulating lysosomal Ca2^+^ homeostasis and receptor recycling, which in turn upregulates chemokine expression, exacerbating fibrosis via the immune-vascular-matrix axis. Furthermore, previous functional studies indicate that TMBIM1 inhibits adipogenesis by destabilizing PPARγ [23, 24], and abnormalities in the PPARγ signaling pathway have been confirmed to participate in the fibrotic pathological process of SSc [25]. Additionally, lipid metabolism disorders are associated with the vascular lesions of this disease [26], hinting at its potential impact on SSc progression through the immune-metabolic axis. PGAP1 encodes GPI-inositol deacylase located on the endoplasmic reticulum membrane, functioning as a crucial “quality control/transformation” link in the maturation of GPI-anchored proteins (GPI-APs) [27, 28]. GPI-AP is ubiquitous on the surface of immune and vascular cells, performing functions such as receptor coordination, complement regulation, adhesion, and migration. CD55/CD59, for instance, are prototypical GPI-AP complement regulatory molecules, and alterations in their expression and localization can markedly affect the complement activation threshold [28, 30]. As a GPI-AP, Thy-1/CD90 regulates the adhesion and migration of fibroblast-endothelial and immune cells and is involved in skin repair and blood perfusion regulation [31]. Research has demonstrated that functional defects in PGAP1 can result in the aberrant expression of GPI-anchored proteins, subsequently impacting cell membrane integrity and signal transduction [32]. Additionally, research has demonstrated that Thy-1 can promote skin fibrosis in SSc [33]. Although no direct literature has reported an association between PGAP1 and SSc thus far, we observed a downward trend of PGAP1 expression, and enriched pathways include extracellular matrix remodeling, immune regulation, inflammatory responses, proteasome activity, oxidative phosphorylation (OXPHOS), and protein homeostasis. Given PGAP1’s pivotal role in the maturation of GPI-anchored proteins, we hypothesize that downregulation of PGAP1 could modify the immune microenvironment and disrupt vascular/matrix interactions in SSc. This occurs through altered surface presentation and impaired signal coupling of GPI-anchored proteins. Furthermore, if the PGAP1-GPI anchor protein axis disrupts complement regulation, cell adhesion/migration, or receptor signaling, it may theoretically contribute to SSc pathogenesis. However, this hypothesis requires further experimental validation. Notably, there is an inconsistency in PGAP1 expression between mouse models and human SSc samples. In human SSc skin tissues, PGAP1 expression is significantly downregulated. In the BLM-induced mouse skin fibrosis model, PGAP1 mRNA levels are also downregulated, whereas PGAP1 levels significantly decreased and then gradually return to normal levels on day 28 in lung tissues after BLM treatment. This discrepancy may stem from several factors. First, the pathological process of the BLM-induced fibrosis model differs fundamentally from that of the chronic, systemic autoimmune disease in human SSc [1]. Second, PGAP1 expression and function are tissue-specific. Finally, as a key enzyme for GPI anchor protein maturation [34, 35], PGAP1 may undergo dynamic functional changes during the fibrosis stage. Moreover, species differences exist in the GPI anchor protein family and related metabolic pathways between humans and mice [32]. Finally, as a key enzyme for GPI anchor protein maturation [34, 35], PGAP1 may undergo dynamic functional changes during the fibrosis stage. Moreover, species differences exist in the GPI anchor protein family and related metabolic pathways between humans and mice [32].

Notably, PGAP1 and TMBIM1 are co-enriched in the OXPHOS pathway. SSc is a fibrotic autoimmune disease marked by the pathological activation of fibroblasts. As the disease progresses, the mitochondrial respiratory function in SSc fibroblasts significantly shifts toward oxidative phosphorylation. The enhancement of oxidative phosphorylation, coupled with glycolytic pathway involvement, collectively constitutes the “metabolic reprogramming” process. This process significantly improves cellular energy generation and biosynthesis, thereby promoting fibrosis [36]. Additionally, abnormal activation of oxidative phosphorylation is closely linked to increased local oxidative stress, which further exacerbates the pathological activation of fibroblasts. Importantly, mitochondrial functional changes associated with oxidative phosphorylation are not confined to fibroblasts [37]. In CD14^+^ monocytes from SSc patients, the increase in mitochondrial DNA copy number is positively correlated with elevated secretion of IL-6. This suggests that changes in oxidative phosphorylation function also play a key role in regulating the pro-inflammatory activation of immune cells [38]. Consequently, the combined action of PGAP1 and TMBIM1 in the OXPHOS pathway may contribute to the onset and progression of SSc.

In our study, single-cell data and subsequent immune infiltration analysis demonstrated the remodeling of cellular composition in SSc skin. Tregs and endothelial cells were markedly decreased, whereas mast cells, lymphoendothelial cells, smooth muscle cells, M1 macrophages, γδ T cells, and activated CD4^+^ memory T cells were increased. M1 macrophages’ polarization is linked to tissue remodeling [39]. γδ T cells rapidly produce IL-17, contributing to inflammatory amplification and fibrotic processes [40]. The downregulation of Tregs suggests inadequate immune suppression [41]. The findings are biologically consistent with the three hallmarks of SSc: microvascular abnormalities, immune dysregulation, and fibrosis. In this study, we observed an upregulation of TMBIM1, which positively correlated with M1 macrophages, γδT cells, and activated CD4^+^ memory T cells, while negatively correlating with Tregs and resting mast cells. PGAP1, on the other hand, was downregulated and exhibited an inverse correlation. The observed inverse correlation pattern aligns with our biomarker section’s mechanism discussion, implying a potential role for TMBIM1/PGAP1 in the immune microenvironment remodeling of SSc. Further verification of their specific functions at the cellular and phenotypic levels is required.

This study revealed that despite a reduction in the proportion of Th17 cells in SSc skin tissue, as indicated by single-cell sequencing data, the differentially expressed genes TMBIM1 and PGAP1 identified from this subpopulation exhibited substantial diagnostic significance. This seemingly contradictory finding actually reflects the fundamental principle in immunopathology: “functional state outweighs cell quantity”. The decrease in the proportion of Th17 cells may precisely reflect their dynamic processes of activation, migration, or terminal differentiation. The remaining Th17 cells in the tissue are likely to be hyperfunctional, accompanied by changes like increased secretion of inflammatory factors, metabolic reprogramming, or epigenetic remodeling. Therefore, their specific transcriptome features can precisely capture the essence of immune dysregulation in diseases and hold the potential to serve as effective biomarkers. Similarly, in studies of other autoimmune diseases, the functional gene profile of key pathogenic immune cells, rather than their absolute quantity, has frequently been shown to be closely associated with disease activity [7, 25, 42]. It is important to emphasize that TMBIM1 and PGAP1 not only exhibit differential expression in Th17 cells but also show significant and consistent expression alterations at the whole-tissue level of SSc patients, as verified in independent datasets. This suggests that both are highly likely to extend beyond the scope of a single cell type and are extensively implicated in key pathological processes, including immune microenvironment remodeling, fibrosis progression, and metabolic disorders in SSc. Hence, the diagnostic significance of these two genes primarily derives from their profound association with the core disease mechanism, rather than merely reflecting the abundance of a specific cell subpopulation.

In summary, our study has elevated TMBIM1/PGAP1 from a mere statistical signal to a “dual hub” with diagnostic stratification and mechanistic insights, offering a verifiable roadmap for precise differentiation and targeted interventions in SSc. However, this study has some limitations. Firstly, the analysis primarily relies on samples from public databases, which generally lack annotations for specific subgroups, such as “systemic sclerosis without scleroderma,” and unified disease staging information. Therefore, the analysis results mainly reflect the overall characteristics of localized and diffuse SSc and may not fully apply to specific subgroups or early disease stages. Furthermore, the conclusion still lacks external validation via independent clinical cohort studies and direct experimental confirmation in the tissues of SSc patients. Secondly, immune infiltration analysis and molecular docking are merely computational inferences, and the specific mechanisms by which the two genes regulate and contribute to fibrosis in Th17 cells have not yet been clarified through functional experiments. Finally, the BLM-induced mouse model cannot fully replicate the complex immune microenvironment of human SSc. Although the diagnostic model performs well in the training set, it has not yet achieved clinical standardization and translational application. Based on the above research findings and the limitations of current studies, future research should focus on the following aspects. Firstly, establish a diverse, high-quality, prospective clinical staging cohort that includes the “no scleroderma” subgroup, early-stage patients, and different types of skin involvement. Combine standardized clinical information with multi-omics data to precisely analyze different subtypes and disease stages for stratified validation. Secondly, we verified the diagnostic value of markers like TMBIM1 and PGAP1 in a multi-center cohort. Additionally, using cell and animal models, we further clarified the molecular networks through which these markers regulate immune metabolism and fibrosis via pathways such as oxidative phosphorylation. Ultimately, we should promote the standardization of relevant diagnostic models for clinical testing and conduct experimental and translational research on targeted therapies based on mechanistic studies, thereby offering new strategies for the early diagnosis, precise classification, and targeted intervention of SSc.

## Supplementary Information

Below is the link to the electronic supplementary material.

ESM 1 (CSV 53.2 KB)

ESM 2 (CSV 20.5 KB)

ESM 3 (CSV 34.6 KB)

ESM 4 (XLSX 62.1 KB)

ESM 5 (XLSX 9.77 KB)

ESM 6 (XLSX 20.1 KB)

ESM 7 (XLSX 17.7 KB)

## Data Availability

The datasets used and/or analyzed during the current study are available from the corresponding author on reasonable request.
